# Supplementary material for: Are carbon emissions trading and green financial instruments synergistic? -Comprehensive quantitative research based on content analysis
Source: PLoS One. 2024 Mar 7;19(3):e0298601. doi: 10.1371/journal.pone.0298601 (PMC10919601; doi:10.1371/journal.pone.0298601)
Supplement: S1 Data — (ZIP) [file pone.0298601.s001.zip › Data/地级市Z_F工作报告.zip!/║■─╧/│ú╡┬╩╨/└┤╫╘╣1⁄2╓┌║┼í╛┬φ┐╦╩2╛▌═°í┐_2018.docx]

各位代表：现在，我代表市人民政府向大会报告工作，请予审查，并请各位政协委员和其他列席同志提出意见。

一、2017年工作回顾

2017年，在省委省政府和市委的坚强领导下，在市人大、市政协的监督支持下，我们认真学习领会贯彻党的十九大精神，落实中央和省委省政府的决策部署，大力实施开放强市产业立市战略，齐心协力，真抓实干，较好完成了市七届人大一次会议确定的目标任务，保持了经济社会平稳健康发展。

1、经济实力持续攀高。地区生产总值达3300亿元（预计数，下同），增长8%左右；一般公共预算收入达249亿元，增长10%，税收收入占地方财政收入比重保持全省领先水平；完成固定资产投资2290亿元，增长14%。

产业扶持有力有效。全面启动并迅速铺开产业立市三年行动计划。出台工业企业降费减负、扶持开放型经济、市长质量奖等政策，发布百亿产业发展基金，安排16.1亿元财政资金支持产业发展，为企业提供过桥贷款4.9亿元、融资担保和小额贷款等65亿元；实施常德品牌行动，品牌农业首站长沙推介活动成果丰硕，签订投资合作项目52个，合约资金近300亿元，有效提升了常德品牌的形象和价值。

新型工业升级壮大。实施园区攻坚大会战，规模工业增加值增长6.5%。开工亿元以上工业项目近100个，鑫曜科技、粤港模科、远大住工二期、石门葛洲坝60万吨白水泥生产线、迪文科技三期等项目建成投产，烟厂易地技改、中联起重机械生产基地、溢多利生物等项目扎实推进。建设标准化厂房139万平方米，完成园区规模工业增加值360亿元，增长10%。常德高新区成功跨入“国家级”方阵，我市成为全省第三个同时拥有国家级经开区和国家级高新区的市州。

现代农业量效齐增。农业总产值达665亿元，增长3.5%。大宗农产品生产保持稳定。国家级农业示范园区达到4个。新增农产品加工企业95家、“三品一标”认证205个。新增粮食大户4158个、农民合作社743家、家庭农场1624家。桃源、鼎城入选中国好粮油行动示范县。澧县获批国家农业可持续发展试验示范区。农村环境整治迈上新台阶，美丽乡村建设全域推进，农民生产生活条件明显改善。

现代服务业加速发展。社会消费品零售总额达1170亿元、增长10.8%。桃花源完成闭园改造正式开园，一批国内旅游新热点加快涌现，旅游综合收入达350亿元。商贸、金融、物流、电商等服务业蓬勃发展，石门获批全国电子商务进农村综合示范县。

2、城乡品质大为改善。着力推进城乡统筹，提升城乡品质。一年来，常德蝉联全国文明城市，获评全国“厕所革命”先进市，入选全国“城市双修”试点城市、全国气候适应型试点城市和全国首批“公交都市”创建城市，城市美誉度和影响力显著提升。

交通体系更加健全。实施交通跨越大会战，完成交通投资120亿元。公路治超持续推进并取得明显成效。新建和改建干线公路146公里、农村公路1536公里，完成危桥改造153座。沅澧快速干线1号大道鼎城岗市至临澧太平段，2号大道石门段、澹水大桥至马堰段，6号大道汉寿段和安乡石龟山大桥建成通车。黔张常铁路、沅水四桥加快推进。安慈高速开工建设。津市窑坡渡、安乡长岭洲码头完成主体工程，陬市千吨级码头启动建设。石长铁路动车开通。全市人民热切期盼的常益长高铁正式获批并开工建设。

功能配套更加完善。实施城镇带动大会战，推进桃花源大桥北端片区、建设桥片区、城东片区、江南片区棚改，全市完成城市棚户区改造49088户。新河水系、穿紫河水系、太阳大道、月亮大道、阳山大道、常德大道西延线、沅江隧道、市民之家、阳明湖片区等重大项目进展顺利，大唐司马城、婚庆产业园、卡乐世界等产城融合工程有序推进。海绵城市建设成果亮相全国“砥砺奋进的五年”大型成就展，成功打造技术示范基地和国际论坛两大品牌。深化智慧城市建设，“我的常德”城市公共服务平台上线运行。城市管理更加精细，县（市）城区建管水平进一步提高，一批城市路网、商业中心、公共服务项目加快建设。津市成功创建国家卫生城市。临澧新安镇入选第二批全国特色小镇。

水利基础更加稳固。完成各类水利工程2.5万处，沅澧“两水四岸”综合整治、水毁修复、小型农田水利、沟渠塘坝清淤等项目取得新成效。实施农村安全饮水巩固提升工程，农村自来水普及率达到89%。成功应对沅水流域特大洪水，确保了人民群众生命财产安全。

能源建设更加完备。滚动修编“十三五”配电网规划，完成电网建设投资10.5亿元，澧州变电站等电力设施竣工运营。酒泉—湖南特高压直流输电工程通电投运。加快新一轮农网改造升级，完成416个村网改造。

3、改革开放纵深拓展。坚持以改革为动力，以开放为方向，凝聚推动常德发展的磅礴能量。

重点改革多点突破。“放管服”、税收征管、事业单位分类、综合行政执法、公共资源交易管理体制等改革成效明显。供销合作社改革、津澧新城国家中小城市综合改革试点稳步实施，农村集体土地确权登记颁证试点、农村集体资产产权制度改革试点形成常德经验。

开放合作不断深化。成功引进中国医药、中国中车、中信、中国建筑、中铁投、北京首创、华大基因、德国卓伯根集团等战略投资者，全年到位内外资761亿元，增长10%。实现进出口总额11亿美元，盐关码头“五定班轮”开通。圆满承办中德法治国家对话研讨会、全运会龙舟决赛、全国100大城市机关党建工作经验交流会、首届海峡两岸旅行商交流大会，成功举办常德旅游节、常德柳叶湖国际马拉松赛。

发展要素有效激活。优化用地和拆迁服务，保障重点项目建设需要。有序推进投融资体制改革，国有投融资公司健康发展。出台实施有利于人才发展政策，评选了一批“常德工匠”“常德技能大师工作室”，尊重人才、崇尚技能的氛围进一步浓厚。

4、民生福祉切实增进。不断加大民生投入，相关领域支出占财政支出达72%；城乡居民人均可支配收入分别达28700元、13900元，增长8.2%、8.6%。综合治理、安全生产、计划生育工作被评为全省先进。常德人民有了更多实实在在的幸福感和获得感。

脱贫攻坚成效显著。实施脱贫攻坚大会战，统筹各类财政资金25亿元，发放扶贫小额信贷4亿元，完成19123人易地扶贫搬迁任务。清理识别不精准贫困人口14.1万人，新纳入8.7万人。年内将有7.5万人脱贫、超100个贫困村退出，石门县脱贫摘帽可期。“扶贫楷模”王新法等一批先进典型争相涌现。

社会保障持续加强。新增城镇就业6.7万人，农村劳动力转移就业4.1万人，发放创业担保贷款2.6亿元。城乡最低生活保障、居民基础养老金、高龄老人生活补贴、孤儿基本生活费标准继续调高，五类社会保险新增参保14.5万人次。城乡低保实现动态管理下应保尽保。完成农村危房改造17654户。成功申报全国居家和社区养老服务改革试点城市。

教育水平稳步提升。国家义务教育基本均衡县创建取得重要进展。改造薄弱学校114所，建设标准化学校82所，新建普惠性幼儿园82所。高考文科平均分连续6年居全省第一，理科平均分连续8年居全省第二。中职教育不断规范，高职教育不断发展。

社会治理不断深化。平安常德建设扎实推进，社会大局和谐稳定，综治民调满意率居全省第二。网格化管理体系更加完善，无上访村（社区）建设全面推进，信访维稳形势持续向好。安全生产基础进一步夯实，未发生重大食品药品安全事故，各类应急突发事件得到全面妥善处置。

各项事业协调发展。湘雅常德医院开业运营，公立医院改革有序推进，基层医疗卫生服务和重大疫病防控不断加强。全市公共文体场所免费开放。计划生育政策全面落实。成功举办市第七届运动会。圆满完成村（居）委会换届选举。统计、外事侨务、住房公积金、民族宗教、对台、侨联、残联、老龄、方志、档案等工作取得新成绩，工会、共青团、妇联作用进一步发挥。

国防动员、国防教育、军事设施保护、人防建设、民兵预备役和双拥工作不断加强。

5、生态保护全面加强。以壮士断腕和背水一战的决心推进环境整治，全面完成中央环保督察交办的290个突出问题整改。

重点整治全面推进。市城区环境空气质量优良天数达276天，优良率达75.6%，同比上升4.9个百分点。构建市县乡村四级河长制责任体系，推进洞庭湖水环境综合治理“五大专项行动”，完成汉寿西洞庭湖保护区核心区5.08万亩欧美黑杨清理，安乡珊珀湖综合治理取得阶段性成果。城乡垃圾一体化问题整治全面完成，城市黑臭水体整治取得实效，省以上考核断面水质达到或好于Ⅲ类水体的比例达90%以上。土壤污染综合防治先行区建设扎实推进。

节能减排取得实效。积极淘汰落后产能，关停整顿“地条钢”企业50余家，完成44家石膏矿山整治，淘汰黄标车3882台，关闭赤峰煤矿、羊耳山煤矿等一批中小煤矿。实施节能技改，推行绿色制造，桃源工业集中区获批省铝循环再生产业园。

生态创建成果丰硕。新造林24.1万亩，义务植树1165万株。成功获得国际湿地城市认证提名资格。津市、安乡、西湖、西洞庭顺利通过全省农村环境综合整治整县推进验收，津市被纳入全国首批农村生活垃圾分类和资源化利用示范县。桃源沅水国家湿地公园、安乡书院洲国家湿地公园通过国家验收。汉寿鹿溪国家森林公园成功获批。穿紫河治理经验经央视深度报道形成全国性影响。

6、政府效能显著提升。切实转变政府职能，获评贯彻落实党中央、国务院重大政策措施的先进典型，获得国务院2017年免督查激励。

依法行政得到加强。自觉接受人大及其常委会的法律监督、工作监督以及政协的民主监督，定期向人大报告工作，向政协通报情况，执行市人大及其常委会的决议决定，积极处理各类审议意见，全年办理人大代表建议343件、政协提案324件。认真吸纳各民主党派和工商联调研成果。出台法治政府建设纲要实施方案，积极开展“七五”普法，全面推行法律顾问制度，严格规范自身行为，政府执行力与公信力不断提升。

政务服务不断提速。在省市媒体公布便民服务电话、涉企收费项目清单等重要事项，建立重点企业联系新媒体渠道、企业服务热线，为企业和群众提供便捷服务。在全省率先推行“二十证合一”，完成行业协会商会与行政机关脱钩任务。开展帮扶工业企业优惠政策落实情况大走访大调查大督导，开展优化经济环境“百科大评议”，实施工程建设、办事服务、行政执法、招投标环境专项整治，发展环境持续改善。

作风形象明显改进。深入开展“两学一做”学习教育，严格落实中央、省委和市委各项作风纪律规定。加强行政监察和审计监督，查处了一批违法违纪案件。进一步压减各类会议、文件、考核评比，“三公”经费同比减少5.8%，政风行风明显好转。

各位代表！过去的一年，是形势逼人、任务艰巨、压力倍增的一年，是励精图治、攻坚克难、努力拼搏的一年，是难中有进、转型跨越、继往开来的一年。成绩的取得，是上级和市委科学决策、正确领导的结果，是市人大、市政协大力监督、鼎力支持的结果，是全市人民众志成城、奋发进取的结果。在此，我代表市人民政府，向积极投身常德经济社会发展的广大干部群众、驻常部队和武警官兵，向各位人大代表和政协委员，向各民主党派、工商联和社会各界人士，向所有关心支持常德发展的同志们、朋友们，表示衷心的感谢！

在看到成绩的同时，我们也要清醒认识到前进道路上的困难和自身工作中的不足：交通区位优势不明显，区域竞争日益激烈；工业经济基础不强，传统产业比较薄弱且复苏困难，新兴产业发展缓慢；产业园区配套不优，实力不强；县域经济、民营经济、开放型经济等短板较为突出，金融在经济发展中的支撑作用还有待增强；脱贫攻坚任务依然艰巨，改善民生与人民日益增长的美好生活需要还有较大差距；政府自身建设还存在不足，干部队伍中不担当、不作为、不会为、不勤不廉的现象仍然存在，经济发展环境还有待进一步优化。对此，我们将高度重视，采取有效措施，切实加以解决。

二、2018年工作安排

党的十九大作出了中国特色社会主义进入新时代的重大判断，向我们释放了强烈而鲜明的政治信号。在这个关键历史节点和崭新发展起点上，我们必须高举习近平新时代中国特色社会主义思想伟大旗帜，紧紧把握全面贯彻落实党的十九大精神开局之年的重大契机，不忘初心，牢记使命，奋力拼搏，开拓进取，用发展实绩将党的十九大精神书写在常德大地上。

进入新时代，我们要勇闯新征程，始终保持发展定力。新时代引领我们踏上新征程。从宏观层面来看，世界经济复苏的不确定性、不稳定性依然存在，我国社会主要矛盾已经转化，但经济长期向好的基本面没有变。面对复杂多变而又利好叠加的新形势、新常态、新征程，我们要保持战略定力和发展耐力，紧紧围绕中央、省委省政府和市委的决策部署，一心一意促发展，一以贯之抓落实。特别是要注重保持战略、工作、政策、措施的连续性和稳定性，做到在坚持中完善、在完善中坚持，促进常德发展步步为营、行稳致远。

进入新时代，我们要顺应新期盼，广泛凝聚发展共识。人民日益增长的美好生活需要和不平衡不充分的发展之间的矛盾是新时代的重要特征，也是我们一切工作的出发点。必须始终坚持以人民为中心的发展思想，把群众对民主、法治、安全、生活、服务、环境等方面的满意度作为工作标尺，不断提升我们的工作水平，切实调动全市人民关心、支持、推动常德发展的主动性和积极性，在顺应群众新期盼中凝聚发展新合力。

进入新时代，我们要践行新理念，切实提升发展质效。新时代常德的发展，应当是以更高质量、更有效率、更加公平、更可持续为新追求的发展。我们务必时刻遵循创新、协调、绿色、开放、共享发展理念，将其践行在创新引领开放崛起和开放强市产业立市战略深化的方方面面，以开放强市所拓展的广阔空间承载质量效益，以产业立市所释放的强大动能激活质量效益。

进入新时代，我们要抢抓新机遇，全力赢得发展主动。党的十九大胜利召开后，壮大实体经济、支持改革创新、实施乡村振兴、促进区域协调、推动全面开放等重点战略全面铺开，长江经济带发展、洞庭湖生态经济区建设等重大利好将进一步向常德释放，为常德跨越崛起创造了优势、拓展了空间。我们要应势而谋、顺势而为、乘势而起，利用自身有利条件加快建设现代产业中心、交通物流中心、科教文卫中心、金融贸易中心和休闲旅游中心，在新一轮区域竞争中抢占发展制高点、打造核心增长极。

根据新的形势和任务，政府工作的总体要求是：坚持以习近平新时代中国特色社会主义思想为指导，全面贯彻党的十九大和中央、省委、市委经济工作会议精神，坚持稳中求进工作总基调，坚持新发展理念，紧扣社会主要矛盾变化，按照高质量发展的要求，统筹推进“五位一体”总体布局和“四个全面”战略布局，坚持以推进供给侧结构性改革为主线，深入实施创新引领开放崛起战略，大力推进开放强市产业立市，在打好防范化解重大风险、精准脱贫、污染防治攻坚战方面取得扎实进展，为建设现代化区域中心城市而努力奋斗。

经济社会发展的主要预期目标任务是：地区生产总值增长8%以上，规模工业增加值增长7.5%以上，固定资产投资增长12%以上，一般公共预算收入增长10%左右，城乡居民人均可支配收入增长8.5%以上，争取全面建成小康社会达标授牌。

为实现上述目标任务，我们将着力推进以下十个方面的工作。

1、着力推进产业行动，培育发展新动能大力推进产业立市三年行动计划，开展“产业项目建设年”活动，进一步优化产业格局，壮大产业实力，提升产业能级，把实体经济的根基筑得更牢、扎得更深。

做强产业园区。科学明晰园区定位和产业特色，推动园区走集约化、特色化、人性化发展道路。按照国家级园区不超过“三主三辅”、省级园区不超过“两主两辅”的思路，专注主业发展，大力发展特色园、园中园，支持创建津澧国家级经开区，年内常德经开区、常德高新区规模工业增加值分别增长10%、12%以上。加强园区水电路气等基础设施建设，完善教育、医疗、娱乐等配套设施与服务，园区基础建设投入100亿元以上，根据需要盘活建设标准化厂房。推动园区去行政化、趋市场化，在选人用人、管理审批、薪酬标准等方面给予更大自主权，增强园区发展内生动力。

壮大产业实力。着力壮大新型工业。认真对接“中国制造2025”、制造强省五年行动计划、全省20个新兴优势产业链和“五个100”部署，针对性建链、补链、强链。全力打造烟草、生物医药与健康食品、装备制造与军民融合、文旅康养四大千亿产业，稳步壮大纺织服装、建材化工、林纸加工等传统产业，加速壮大新能源、新材料、信息技术等战略性新兴产业。力争烟厂易地技改、中车新能源汽车、中联起重机械生产基地等项目建成投产，加快建设石墨烯新材料、藏格集团复合肥生产线等项目。积极培育百强核心骨干企业，大力实施中小微企业成长工程，新增10亿元企业3家以上、亿元企业30家以上、规模工业企业100家以上。着力壮大现代服务业。发展全域旅游，加快推进卡乐世界、大唐司马城等项目建设，持续办好旅游节庆活动，大力发展城市旅游、乡村旅游、红色旅游、生态旅游，支持石门创建国家全域旅游示范县，打造一批知名旅游景区和精品旅游线路，加快推进旅游与文化、体育、康养等产业融合发展，全年旅游总收入增长20%以上。发展现代金融，支持各类金融机构引进与发展，加快建设柳叶湖基金小镇，举办全国性基金峰会。加强政府债务管理，防范区域金融风险。发展现代物流，力争传化公路港物流等项目开工建设，抓好物流标准化城市试点，形成便捷畅通的城乡物流网络。引导传统商贸企业创新商业业态和模式，进一步提升城市商圈品质和人气。高起点建设文化创意产业园，大力发展电子商务、服务外包、数字经济等新业态。促进房地产业健康发展。社会消费品零售总额增长10%以上。

打响产业品牌。大力实施常德品牌行动，提升“常德品牌、中国品质”影响力。定期举办常德品牌产品推介活动，支持企业参加各类境内外展会，与知名电商、龙头企业、大型商场开展产销对接、投资合作。扎实推进质量强市建设，引导全社会强化品牌意识，弘扬工匠精神，培育一批品牌产品、品牌企业、品牌工程。新增中国驰名商标2件以上、马德里国际注册商标5件以上、农产品“三品一标”认证100个以上；全力推进中国旅游休闲示范城市创建，桃花源力争创建国家5A级旅游景区，柳叶湖力争创建国家级旅游度假区。

强化产业扶持。全面落实降费减负、产业发展、扶持开放型经济等政策措施，降低要素成本。引导金融机构服务实体经济发展，新增银行贷款200亿元以上、表外融资200亿元以上；引导企业对接资本市场，实施企业上市（挂牌）五年行动；突出财政性资金扶持重点，用好用活产业发展基金，扶持特色园区和主导产业发展。盘活土地资源，优先保障重点产业项目用地需求。切实加强企业帮扶，大力实施“暖企”行动，加大本地产品使用支持力度，建立健全企业服务平台，充分发挥企业服务热线、重点企业联系新媒体渠道作用，及时有效解决企业困难。

开放强市产业立市，离不开企业的市场主体作用，离不开企业家的智慧和创造。各级政府及其相关部门要舍小利、顾大局，扶持企业成长，保护企业家权益，以“亲”的情感服务企业，以“清”的原则要求自己。在沅澧大地上，我们一定要让办企业被尊重，让做实业受追捧，让投资者得实惠，让创业者有成就！

2、着力推进对外开放，拓展发展新空间

突出精准招商。强化招商引资责任，坚持主要领导带头抓招商，分管负责人集中主要精力抓招商，驻外机构、工业园区全力抓招商。创新招商引资方法，把握投资导向、跟进投资动向、瞄准前沿方向，开展定向性、跟进式、精准化招商，与发达地区的园区、商会和企业结对合作，积极开展“迎老乡、回故乡、建家乡”行动。突出招商引资重点，主攻港澳台、长三角、珠三角、京津冀等重点区域，积极对接世界500强、中国500强和民营500强，深化与央企省企的战略合作，鼓励发展总部经济。各区县（市）和常德经开区、常德高新区全年落地开工的亿元以上产业项目不少于15个，其中工业项目不少于7个。

提升开放平台。积极构建水、陆、空立体化口岸体系，突出抓好德山水运口岸建设，支持常德—上海（岳阳）“五定班轮”常态化运营，组建常德港务集团。积极拓展航班航线，力争新增航线不少于3条，每周航班总数不少于50个，稳步开展航空临时口岸申建工作，力争年内开通国际包机。申报建设常德保税物流中心（B型），大力发展保税经济。加强“单一窗口”和口岸信息化建设，降低通关成本，提升通关便利化水平。

促进“三外”联动。推进外经、外贸、外资融合发展。深入实施“破零倍增”和业绩回流行动，做大外贸经济总量。大力实施德企德品出海行动，推动优势产业、优秀企业、优质产品走出去。着力推动加工贸易转型升级，大力发展服务贸易，培育发展贸易新业态。积极支持优势行业、进出口企业对接跨境电商平台，开拓国际市场。实现进出口总额13亿美元以上，增长20%以上。

常德作为不靠海、不沿边的内陆城市，我们一定要有兼容并蓄的姿态、勇于担当的精神、高效务实的行动，坚持主动开放、双向开放、全面开放，努力打造开放包容的常德形象！

3、着力推进改革创新，增强发展新活力

扎实推进改革攻坚。以供给侧结构性改革为主线，加大“三去一降一补”力度，深化“放管服”改革，下放行政权限，创新监管体制，减少行政审批，推进生态环境监管体制、社会领域等重点领域改革。加大国有企业改制力度，推进“三供一业”分离移交。推进农村土地制度改革，完善承包地“三权”分置制度。推进园区管理体制、口岸综合管理协调机制等一批自主改革，构建创新型经济新体系。

大力推动科技研发。强化企业创新主体作用，加强产学研用紧密结合，推动产业链、创新链、资金链深度融合。组织开展“智汇洞庭、科创未来”科技成果系列展示对接活动，推动科技成果转化。推进大众创业万众创新，建设一批双创示范基地，支持大学生、专业技术人员、返乡务工人员创新创业。年内新增国家级和省级科技企业孵化器各1家以上、省级星创天地2家以上，新认定高新技术企业20家以上，高新技术产业增加值达到350亿元，增长15%。强化知识产权创造、保护与运用。推动“两化”融合、军民融合。

致力人才机制创新。坚持多方引才、精心育才、大胆用才、环境留才，打造区域性人才高地。加大各类优秀人才引进力度，给予相关补贴和待遇。实施本土人才培育工程，年内培训高技能人才2000人以上，培育一批“常德企业家”“常德工匠”“常德能人”。完善人才柔性流动机制，在子女教育、家属就业、社会保障、住房保障等方面给予倾斜，以强大的环境软实力让本地人才留下来，域外人才走进来，各类人才聚拢来。

4、着力推进乡村振兴，夯实发展新基础

按照产业兴旺、生态宜居、乡风文明、治理有效、生活富裕的总要求，促进城乡融合发展，加快推进农业农村现代化。

立足提质增效，大力发展现代农业。推进农业供给侧结构性改革，推动粮食、油菜、水果、蔬菜、茶叶、林业、油茶、养殖等主导产业转型升级。抓好西湖西洞庭和桃源国家现代农业示范区、国家农业科技园区和各类特色农业专业园区建设。支持农业产业化龙头企业发展，做大做强农产品加工业，大力促进农业与工业、文化旅游、生态休闲、现代物流等二三产业融合发展，积极建设田园综合体，延伸产业链，提升价值链，增强竞争力。

立足搞活经营，大力培育新型主体。加强新型职业农民培育，壮大新型农业经营主体，年内新增农民合作社500家以上、家庭农场1000家以上。健全农技推广、疫病防控、农机作业等社会化服务体系。全面完成农村土地确权登记颁证，引导土地有序流转，发展多种形式适度规模经营。深化农村集体产权制度改革，探索农村土地经营权抵押贷款。深化供销合作社综合改革，提升为农综合服务水平。大力发展农村电商，搞活农产品流通。

立足固本强基，大力提升设施条件。全面落实强农惠农富农政策，增强村级集体经济造血功能，促进农民收入稳定增长。落实国家粮食安全战略，加强农田水利设施建设，开展农村土地综合整治，加强优质粮油示范工程建设，推进粮油生产机械化，提升现代农业综合生产能力。发展农业政策性保险，提高农业抗风险能力。做好防汛抗旱、森林防火、动植物防疫等工作。

立足生态宜居，大力建设美丽乡村。深入实施美丽乡村建设行动计划，精心建设一批各具特色、尽显其美的美丽乡村、美丽庭院。提升农村基础设施现代化水平，抓好农村公路、信息网络的升级改造，倡导推进“厕所革命”。丰富农村文化生活，抓好移风易俗，倡导文明乡风，完善农村公共服务。

5、着力推进城镇带动，构建发展新格局

以城市拓展和改造为抓手，精心谋划、精明规划、精细建管，推进城镇品质提升。优化城市空间布局。完成《常德市总体城市设计》报批，编制完善近期规划和各类专项规划。

优化城市空间结构布局，做好北部新城、西部新区、东部城区、江南新城、德山新城等片区建设发展，推动一江两岸协调发展。依法强力控违拆违，保障城乡规划顺利实施。

提升城市功能品质。持续推进海绵城市建设，大力发展海绵产业，创建一批海绵示范工程，确保通过三年海绵城市试点考核验收。全力推进“城市双修”十大工程，启动国家生态园林城市创建申报，加强柳叶湖及周边生态环境保护。推进城市棚改、高铁新城、沅江隧道、沅水五桥、奥体公园、会展中心、第一工人文化宫等重点项目建设。结合常德传统文化元素，打造一批特色建筑和装配式建筑示范工程。健全城市管理和执法机制，推进城市管理向基层下移，建立完善责权明确、运行有序、条块结合的大城管工作体制。大力推进“公交都市”创建，规范传统出租车和网约车管理。加大城市地下空间利用力度，加强公共停车场建设。

促进城乡协调发展。抓好县城规划发展，推进县城品质提升，提高县城在县域经济中的首位度。稳步推进津澧融城、常桃融合，打造市域副中心。抓好津澧新城国家新型城镇化试点和中小城市综合改革试点、临澧产城融合试点。实施“两西”发展战略，打造常德卫星城。抓好新型城镇和特色小镇建设试点，打造现代化城镇群。加快农业转移人口市民化进程。

6、着力推进县域发展，壮大发展新引擎

实施扩权强县。扩大县级经济社会管理权限，切实提高县级抓发展的自主权、主动性和积极性。扎实推进石门县省直管县经济体制改革试点，积极推进经济发达镇行政体制改革，探索推进市、县事权与支出责任划分改革。加快转变乡镇政府职能，加强乡镇政府服务能力建设。

强化引导支持。统筹规划和支持各区县（市）产业项目建设，形成特色鲜明、布局合理、差别竞争、优势明显的产业格局。鼓励支持各区县（市）设立产业发展基金。谋划推进有利于带动县域发展的基础设施项目，加快构建市域内快速通勤圈，打通县域交通内循环。在资金、土地等方面对区县（市）予以倾斜，助推县域经济转型升级、做大做强，力争有更多县（市）进入全省县域经济二十强。

重视基层基础。加大乡村社区运转经费保障力度，改善基层工作生活条件，保障好乡镇、村居干部待遇。重视和关心基层干部，鼓励优秀人才在基层建功立业、成长成才。规范和精简面向基层的检查考核、评比达标活动，切实为基层减轻压力。

7、着力推进基础建设，打造发展新高地

实施重点建设项目300个，完成投资900亿元以上，全力推进100个重大项目、200个重大前期项目。

加快交通枢纽建设。继续加大交通建设力度，完成投资120亿元以上。加快黔张常铁路、常益长高铁建设，力争襄常高铁、常德铁路交通枢纽站开工建设。加快安慈高速建设，开工建设常益高速公路复线、官庄至新化高速公路，推进宜石张高速前期工作。确保沅水四桥、津市澧水二桥、安乡长岭大桥、石门杨岭岗澧水大桥年内通车或完成主体工程，改造病危桥100座，统筹规划建设好千吨级码头。加快沅澧快速干线建设，确保2号大道临澧段、澧县段、4号大道河洑至桃源段、6号大道常德经开区段建成通车，启动和加快推进其它路段建设。完成沅水澧水航道整治工程。新建改建干线公路120公里以上，确保319、207国道市城区改线工程建成通车。完成农村公路窄改宽工程653公里、通自然村公路2800公里。建立健全公路管理养护长效机制，推进治超常态化。

加快水利设施建设。全年完成水利投资59亿元。加快完善防洪安全网、城乡供水网、农业灌溉网、水生态保障网和水利信息网。推进江河湖库连通、安乡澧水片区饮水工程、黄石水库水资源综合利用、重点垸堤防加固、大中型水闸和小型病险水库除险加固等工程建设，抓好大中型灌区续建配套、大型泵站更新改造、沟渠塘坝清淤增蓄等农田水利建设。大力实施农村安全饮水巩固提升工程。

加快能源基础建设。启动海德、高丰输变电工程。抓好电网规划滚动修编。积极推进农村电网改造升级，完成530个村网改造。支持发展风电、太阳能、地热能和生物能等新能源，抓好汽车充电桩建设。

加快智慧常德建设。加快创建国家新型智慧城市建设试点城市。深化政府数据资源共享，建成网上政务大厅一期和“我的常德”城市公共服务平台二期项目。扎实推进重点应用系统建设，大力发展“互联网+”经济，培育发展大数据、云计算、物联网产业，加快发展武陵区移动互联网产业园和德山电子信息产业园。

8、着力推进生态保护，厚植发展新优势

加强生态系统保护。统筹推进山水林田湖草的系统保护。严守耕地保护红线，加强矿产资源保护性开发和高效利用，抓好地质灾害防治工作。推进国际湿地城市建设，开展国土绿化行动，推动退耕还林还湿。健全生态环境保护责任体系，建立领导干部自然资源资产离任审计制度。

深化环境综合治理。持续推进中央环保督察整改、洞庭湖水环境综合治理，强力整治突出环境问题。开展全国第二次污染源普查工作。全面落实河长制，启动实施湖长制，协同推进河湖管理保护和生态环境治理，禁养区内规模化畜禽养殖全部退出，完成县级以上饮用水水源保护区规范化建设。新建改建6个污水处理厂，全市污泥无害化处理率达100%。编制大气污染源排放清单，加强餐饮油烟、秸秆焚烧、工地扬尘等污染源治理，促进市城区环境空气质量优良天数稳步提升。全面推进土壤污染综合治理，完成土壤环境质量详查。出台城乡垃圾处理地方性法规，加快城乡垃圾一体化治理。强化企业主体责任，加强环境督察联动执法，曝光一批破坏生态环境的违法行为和案件。

推进绿色循环发展。建立资源总量和全面节约制度，推动重点行业整治整合，完成省下达的节能减排任务。积极培育节能环保、清洁能源等新兴产业，大力发展循环农业，减少面源污染。开展循环经济示范行动，倡导低碳出行和绿色生活方式。

9、着力推进民生改善，共享发展新成果

坚决打赢脱贫攻坚战。全市贫困村退出100个以上，脱贫5万人以上，确保石门县脱贫摘帽。坚持精准识贫、精准扶贫、精准脱贫，抓好健康扶贫，强化建档立卡贫困人口“五重医疗保障”，建立健全贫困助学帮扶机制，完成实用技术培训、危房改造、易地扶贫搬迁等工作任务，加强贫困村水电路网等基础建设，建立“新型经营主体+贫困村+贫困户”的产业扶贫模式，为贫困人口构建更加紧密的利益联结机制，做到脱真贫、真脱贫。

优先发展教育事业。促进教育优质发展。全面启动标准化学校和新一轮市城区中小学（幼儿园）规划建设，培养高素质高水平教师队伍，切实提高教学质量，优化教育环境，确保校园安全。促进教育协调发展。支持发展职业教育和高等教育，加强职教大学城建设管理，推进常德财经职院（筹）新校区建设，改善县级公办职中办学条件。抓好民办教育、特殊教育、网络教育和老年教育。促进教育公平发展。统筹城乡义务教育一体化改革，大力改善农村学校和薄弱学校办学条件，加快发展公办和普惠性民办幼儿园，严格执行阳光招生，确保每个孩子有学上、上好学，都享有公平接受教育的权利。

积极发展卫生健康事业。深化医药卫生体制改革，积极探索城市公立医院改革试点，加强基层医疗卫生服务体系建设，重点推进市一中医院、市妇幼保健院等项目建设，完善湘雅常德医院周边配套和运营管理。全面深化计生优质服务，加强出生缺陷综合防治，严格落实奖励扶助政策。深入开展爱国卫生运动。预防控制重大疾病、突发急性传染病和慢性病。积极开展国家精神卫生综合管理试点。坚持中西医并重，传承发展中医药事业。支持和规范社会办医。推进居家和社区养老服务模式试点，探索医养结合新模式。加快建设中国中药常德产业园、医药康养产业园。

大力促进就业创业。推进创业带动就业三年行动计划，抓好高校毕业生、农村转移劳动力、城镇困难人员等重点群体就业，完善创业孵化基地运行机制。落实劳动保障重大违法行为社会公布和诚信评价制度，构建和谐劳动关系。加强职业技能培训，强化职业院校与本地企业合作，缓解企业“招工难”问题。新增创业主体2.7万户，新增城镇就业5.6万人、农村劳动力转移就业3.5万人。

加强社会保障体系建设。全面实施全民参保计划，五类社会保险新增扩面12万人次以上。完成机关事业单位养老保险制度改革，抓好企业养老保险缴费费率过渡试点，深入推进城乡居民医保制度整合和异地就医联网结算，巩固完善工伤保险和大病保险政策机制。完善社会救助保障制度，稳步提高城乡低保水平。关爱失独家庭、农村留守人员，重视老龄事业发展，加强残疾人康复服务，发展残疾人事业。

繁荣文化体育事业。完善现代公共文化服务体系，传承发展优秀传统文化，做好文物保护修缮工作，支持城头山古文化遗址、万里茶道石门段宜红古茶道申报世界文化遗产。实施文艺精品工程，推动文化市场和文化产业健康有序繁荣发展。办好各类群众性文化活动，广泛开展全民健身运动，办好常德柳叶湖国际马拉松赛等重大赛事。

协调发展其它事业。做好粮食、外事侨务、民族宗教、防震减灾、对台、方志、气象、档案、保密等工作。认真开展第四次全国经济普查。加强住房公积金归集扩面和资金管理。支持工会、共青团、妇联等群团组织工作。加强全民国防教育，建设智慧动员体系，严格落实双拥优抚安置政策，抓好军事设施保护、人民防空、民兵预备役和兵役征集工作。

10、着力推进社会治理，营造发展新环境

化解矛盾纠纷。深化信访制度改革，压实信访工作责任，完善领导干部公开接访、依法处访、妥善息访、信访终结等工作机制。抓好矛盾纠纷源头治理，深入开展无上访村（社区）建设。加强和改进社区矫正、法律援助、司法鉴定等工作，推动公共法律服务城乡全覆盖，健全人民调解、行政调解、司法调解联动工作体系，构建矛盾纠纷多元化解渠道。

维护公共安全。全面推进国家安全发展示范城市创建，完善安全生产责任制和考核体系，加强安全生产监管能力和智慧安监建设，深化安全生产专项整治和事故隐患排查治理，坚决遏制较大及以上安全事故。创建国家食品安全城市，抓好五大体系和十大工程建设，深化食品药品重点对象、重点场所、重点产品专项整治，确保“舌尖上的安全”。深入开展平安创建活动，依法严厉打击黑恶犯罪、毒品犯罪、涉众性侵财犯罪等各类违法犯罪行为，提高突发事件的应急处置能力，不断巩固综治民调成果。

完善治理体系。打造共建共治共享的社会治理格局，健全网格化服务管理和综治中心一体化机制，完善农村综合服务平台，不断提升基层组织治理能力和水平。加强社会信用体系建设，推进市县信用信息共享交换平台一体化，构建失信联合惩戒机制。健全社会组织管理制度。大力推行“互联网+社会治理”模式，加强治安防控体系建设，推进雪亮工程，实现城区和骨干道路视频监控探头全覆盖并向乡村延伸。二号首长

三、加强政府自身建设

初心激发使命，使命呼唤担当。新时代、新目标，必须有新气象、新作为，各级政府要驰而不息加强自身建设，强化责任担当，增强施政本领，提高行政效能，服务全市人民。

1、坚持亲民利民，做到为民行政。深刻认识我国社会主要矛盾发生的重大转变，始终坚持以人民为中心的发展思想。牢固树立群众观念，站在群众立场看问题、想问题、解决问题，坚决纠正思维惯性、路径依赖和政绩观偏差。认真践行群众路线，坚持问政于民、问计于民、问需于民，主动到基层去，到人民群众中去，特别是到问题最突出、矛盾最集中的地方去，真诚倾听群众呼声，真实了解群众愿望，真情关心群众疾苦。切实维护群众利益，始终牢记群众利益无小事，着力在增加居民收入上下功夫，在办好民生实事上抓深入，在解决民生难题上求突破，在服务群众上增效能。深化行政审批制度改革，优化审批流程，减少审批环节，继续推进“互联网+政务服务”，在园区推行全程代办、一网通办、在线办结等服务方式，实行政务大厅之外无审批、公共服务事项全覆盖的运行模式，打通服务群众“最后一公里”。

2、坚持认真较真，做到务实施政。认真才能担事干事，较真才能动真碰硬。坚决说到做到。增强狠抓落实本领，说实话、办实事、出实招、求实效。说了的坚决做到，定了的坚决执行，对重大决策、重点项目、重要工作挂图作战、压茬推进，加大工作交办、催办、督办力度，真正做到件件有着落、事事有回音。力戒形式主义。坚持一切从实际出发，多做打基础、利长远的事情，不搞花架子，只下实功夫。调查研究做到轻车简从、简化接待、注重实效。进一步转变文风会风，切实减少各类会议，切实精简文件简报，开短会、讲短话、发短文，坚决防止以文件落实文件、以会议落实会议，能不开的会议坚决不开，能合并的会议坚决合并，可发可不发的文件坚决不发。推行一线作为。大力践行“一线工作法”，让干部在一线工作，领导在一线指挥，问题在一线解决，能力在一线提升，绩效在一线考核，奖惩在一线兑现，努力做出经得起实践、人民和历史检验的工作实绩。敢于攻坚克难。切实强化责任担当，勇于直面问题，敢于迎难而上，做到矛盾面前不回避、困难面前不退缩、责任面前不推诿，下大力抓好具体工作推进、具体问题解决，完善为担当者担当的体制机制，激励干部释放担当的激情和能量，推动形成勇于干事创业的良好局面。

3、坚持公开公正，做到阳光理政。只有公开才有监督，只有公开才有公正，只有公开才有公信。围绕服务发展推进公开，加大对支持企业和园区发展优惠政策的公开力度，更好地支持企业和实体经济发展。围绕民生改善推进公开，全面公开脱贫攻坚、社会救助、就业创业、环境保护、项目和专项资金使用等与群众切身利益相关的信息，主动接受群众和社会监督。围绕规范执法推进公开，重点推进企业生产经营、产品质量、安全生产、公共资源交易行为等方面的监管执法信息公开，以公开促进政府公正执法、企业规范运行。围绕工作落实推进公开，及时公开重点工作进展情况、各项任务指标完成情况，形成比学赶超、真抓实干的浓厚氛围。

4、坚持尊法守法，做到依法行政。依法行政，建设法治政府，是全面落实依法治国基本方略的重要内容，是政府施政的基本准则。增强法治意识。带头尊法学法守法用法，不触碰法律红线，不逾越法律底线，着力营造办事依法、遇事找法、解决问题用法、化解矛盾靠法的法治氛围。规范行政行为。严格按照法定权限和程序依法决策、科学决策、民主决策。提高政府立法质量，构建完备立法体系。深化行政执法体制改革，严格依法履职用权，规范公正文明执法，对破坏经济环境的人和事绝不姑息。自觉接受监督。自觉接受市人大及其常委会的法律监督和工作监督，执行市人大及其常委会的决议决定，认真研究处理各项审议意见，主动接受市政协的民主监督，扎实办理人大代表建议和政协提案，虚心听取和采纳各民主党派、工商联和无党派人士的意见建议，重视舆论监督和社会监督，让权力始终在阳光下运行。

5、坚持清正清廉，做到干净从政。只有干部清正，政府才会清廉，政治才会清明。要严明纪律规矩。坚决维护以习近平同志为核心的党中央权威，坚持把政治纪律和政治规矩挺在前面，持之以恒推进正风肃纪，确保政令畅通、风清气正。要落实廉政责任。认真落实全面从严治党要求，严格履行主体责任和“一岗双责”，推进谈心谈话常态化，切实把廉政责任扛在肩上、抓在手中、落在实处。要扎牢制度笼子。坚持以制度管权、管人、管事，以“亲”上加“清”为原则建章立制，加快构建亲而有度、清而有为的新型政商关系。加强财政资金支出、工程项目招投标、公共资源交易等重点领域廉政风险防控体系建设，强化审计监督，从源头堵住廉政风险漏洞。落实监察体制改革，不断铲除滋生腐败的土壤，严厉惩治各类腐败行为，坚决整治“雁过拔毛”等发生在群众身边的不正之风，以实际行动、清廉作风赢得人民群众的信任和支持。

各位代表，新时代的宏伟蓝图已经绘就，新时代的伟大征程已经开启。让我们更加紧密地团结在以习近平同志为核心的党中央周围，在市委的坚强领导下，大力推进开放强市产业立市，锐意进取，奋力开拓，为建设现代化区域中心城市、决胜全面建成小康社会而努力奋斗！
